# Cutaneous granular cell tumor presenting as a solitary preauricular nodule: a case report

**DOI:** 10.3389/fmed.2025.1689453

**Published:** 2025-11-17

**Authors:** Jialu Song, Xiaomei Han, Lu Zhao, Yi Cheng, Caixia Hu

**Affiliations:** The Fourth Hospital of Hebei Medical University, Department of Dermatology, Shijiazhuang, China

**Keywords:** granular cell tumor, cutaneous, face, preauricular, immunohistochemistry, S-100 protein, CD68, dermatopathology

## Abstract

A 32-year-old female patient visited our hospital due to a solitary firm nodule in the right preauricular area for 4 years. Histopathological manifestations of the skin were nested tumor tissue in the dermis, with polygonal tumor cells, which had abundant cytoplasm filled with eosinophilic granules, and were stained pale pink. Immunohistochemical markers like S-100, vimentin and CD68 were positive. She was diagnosed with cutaneous granular cell tumor and treated by complete resection.

## 1 Introduction

Granular cell tumor is named for its morphological manifestation of substantial eosinophilic granules contained in the cytoplasm. This rare tumor primarily affects adult women (60%), with a mean age at diagnosis of 45. 8 years (1), typically presenting as solitary, reddish-brown or skin-colored subcutaneous nodules. Although usually asymptomatic and benign, it can cause pain, has a tendency for local recurrence, and may rarely undergo malignant transformation with multiple tumors or distant metastasis. This report of a 32-year-old female with a solitary facial nodule highlights the diagnostic challenge of such lesions and informs the differential diagnosis.

## 2 Case report

**FIGURE 1:**
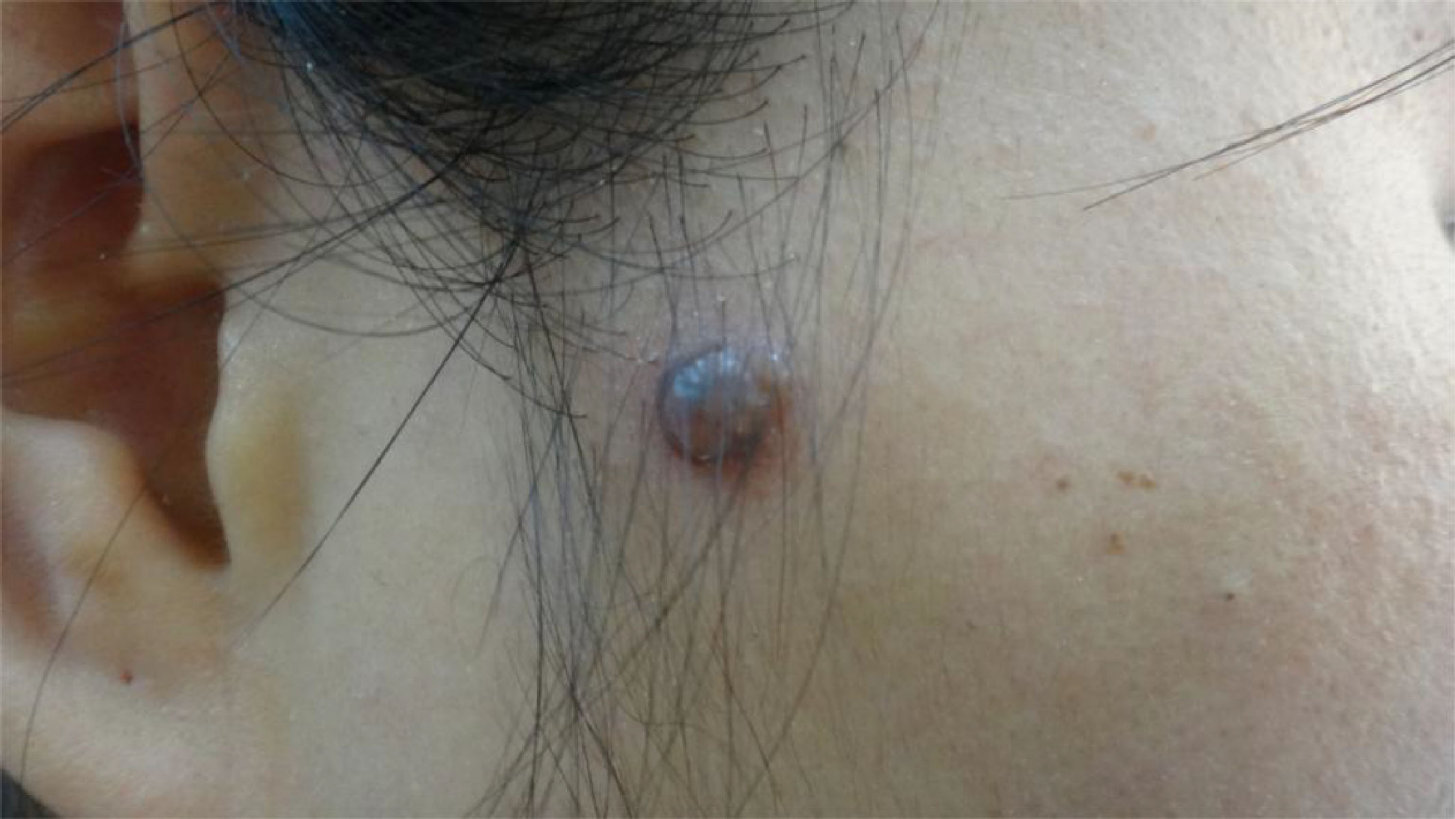
Clinical presentation of a brown, solitary nodule in the right preauricular area.

**FIGURE 2:**
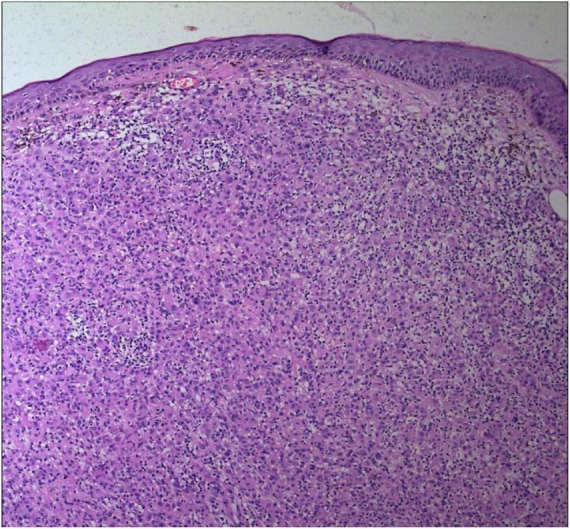
The epidermal processes disappeared, and the dermis was diffusely infiltrated by substantial tumor cells that were in the nested or clumpy form (Hematoxylin and Eosin Stain, × 100).

**FIGURE 3:**
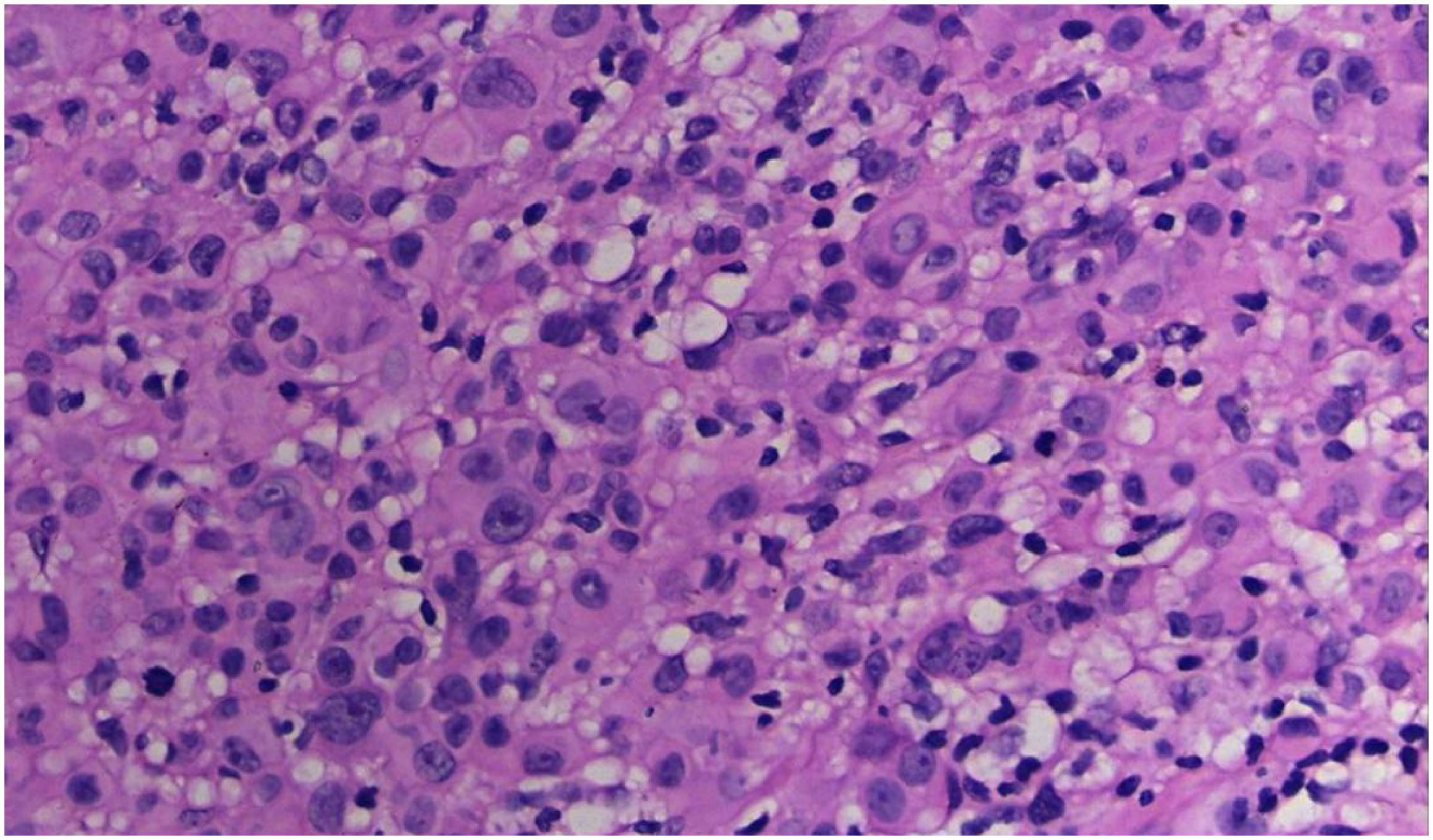
The tumor cells were polygonal, either round or oval, which had abundant cytoplasm filled with eosinophilic granules, and were stained pale pink. The nuclei were small, hyperchromatic and centered, with few mitotic figures, and multiple nuclei were observed in some tumor cells (Hematoxylin and Eosin Stain, × 200).

**FIGURE 4:**
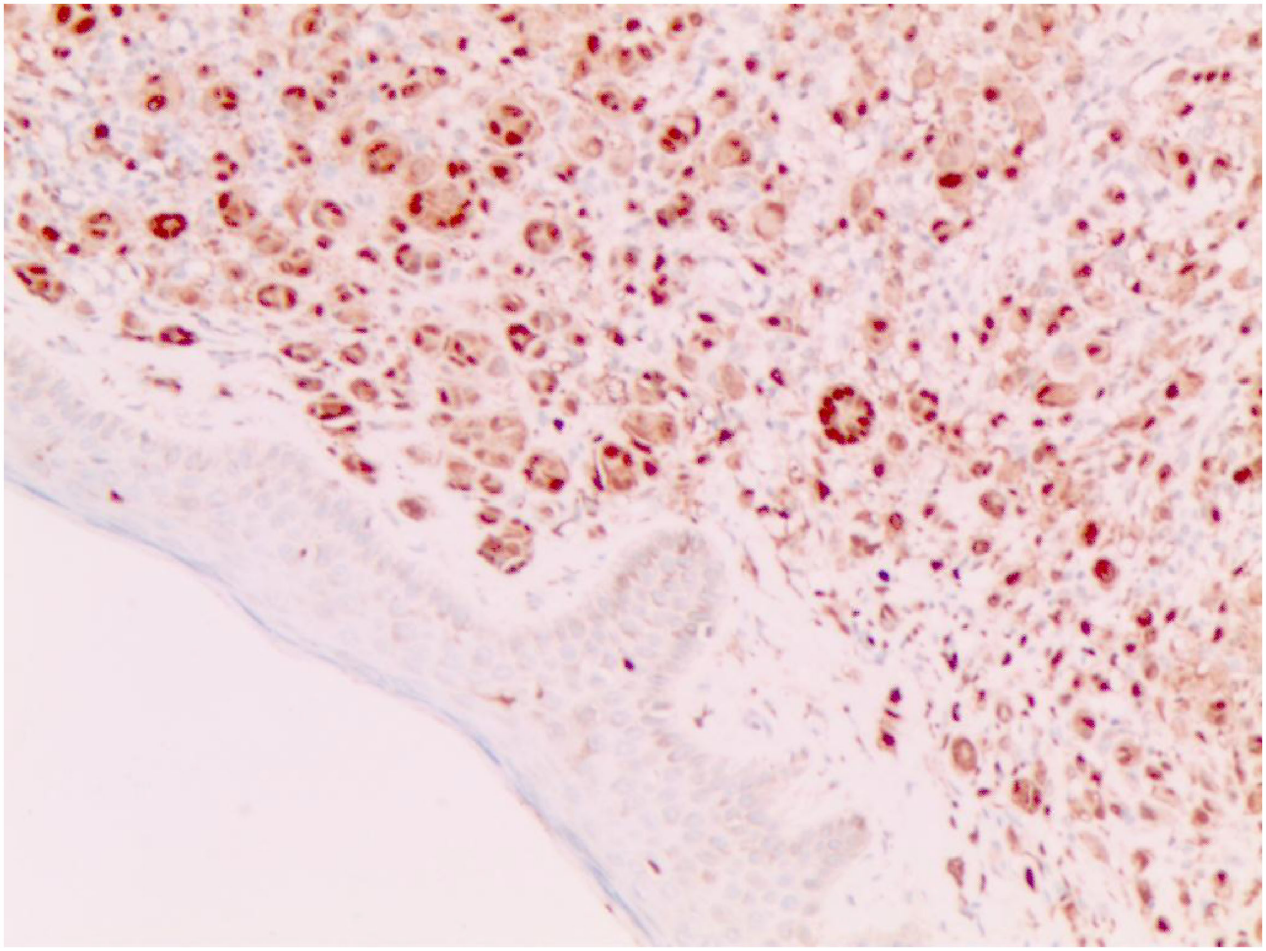
S-100 (+) (Immunohistochemical staining, × 200).

**FIGURE 5:**
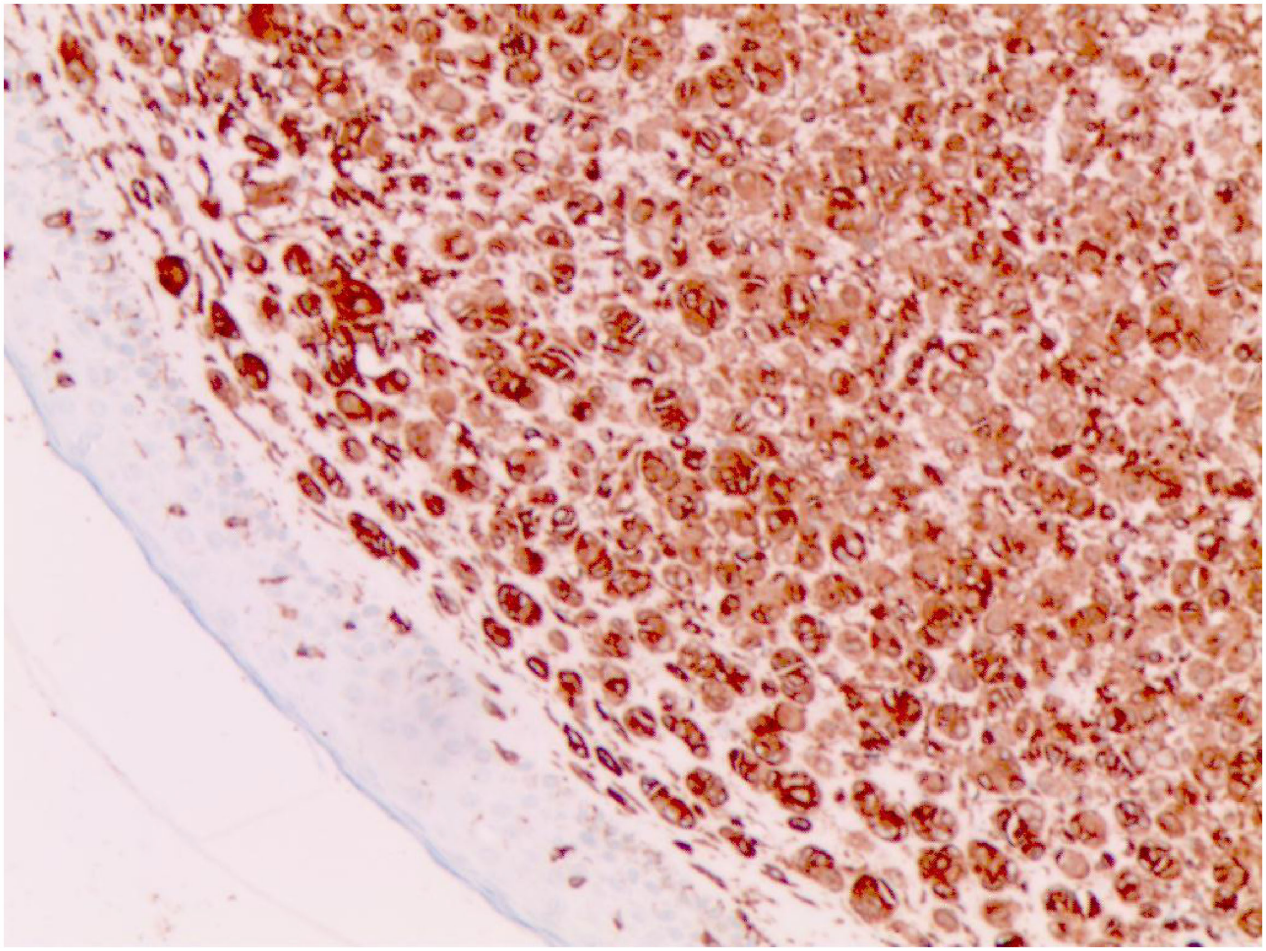
Vimentin (+) (Immunohistochemical staining, × 200).

**FIGURE 6:**
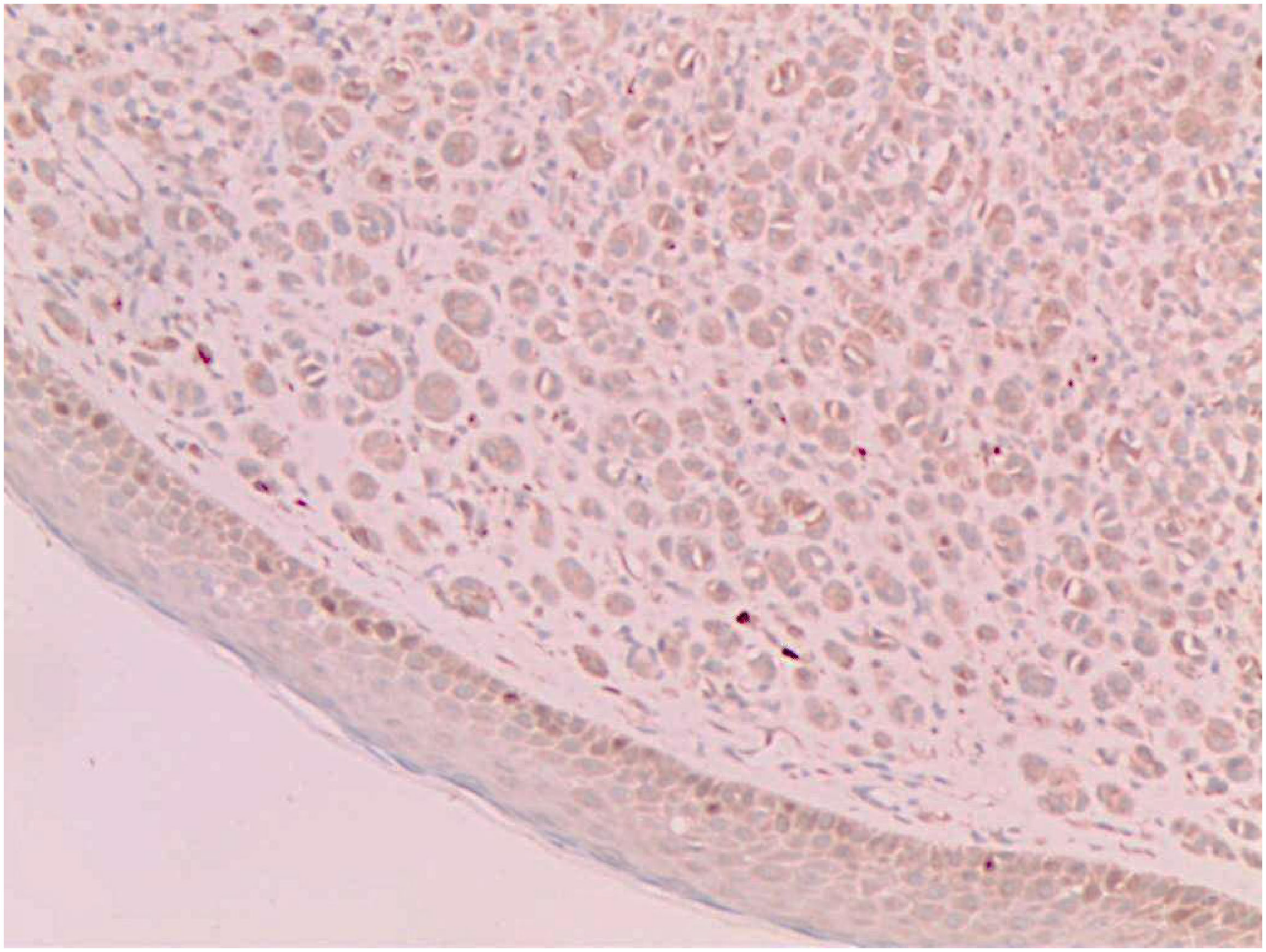
CD68 (+) (Immunohistochemical staining, × 200).

**FIGURE 7:**
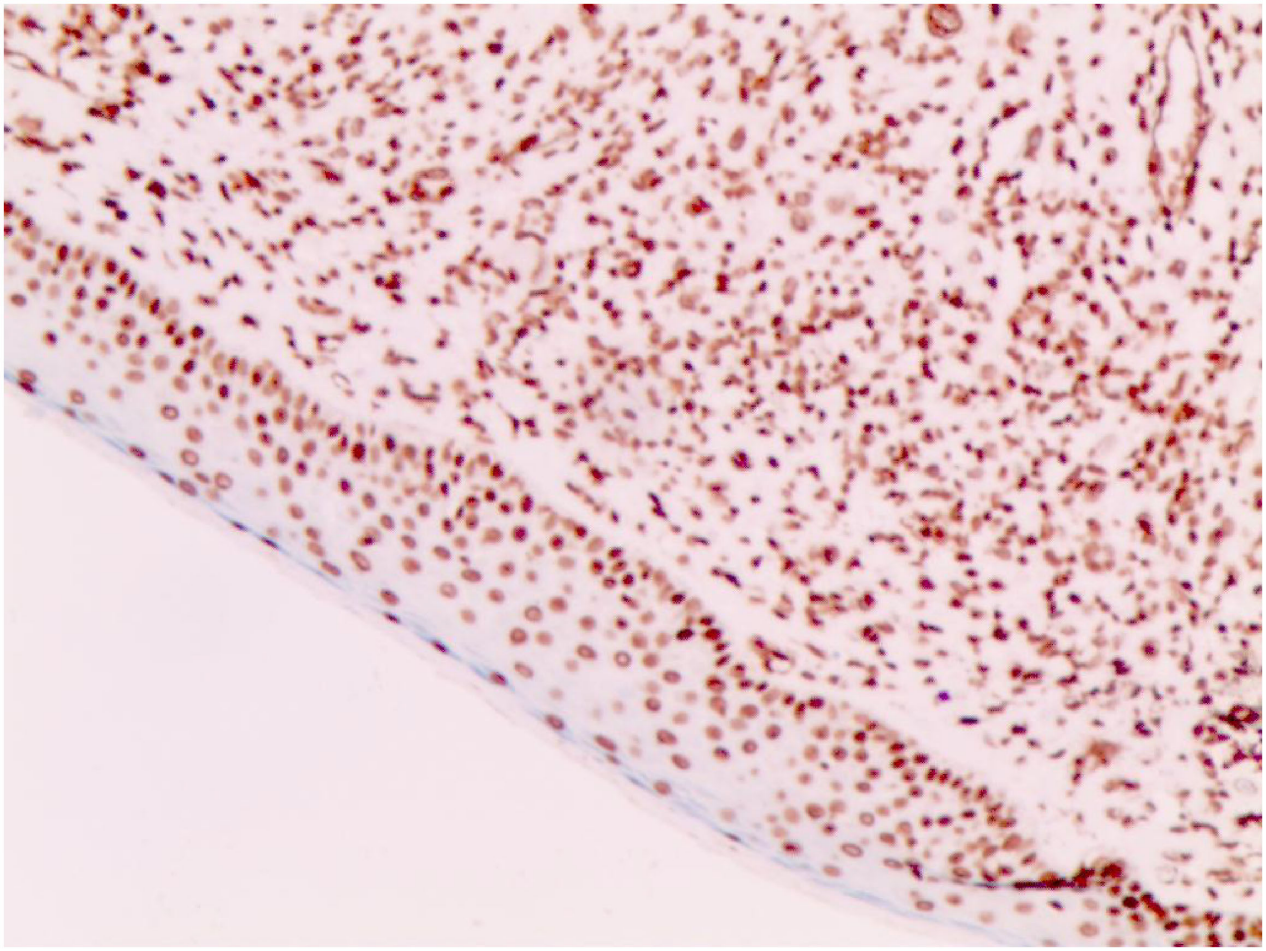
SAM (+ –) (Weakly positive, Immunohistochemical staining, × 200).

A 32-year-old female patient visited our hospital due to a preauricular nodule on the right face for 4 years and pruritus for 1 week. Four years ago, she developed a rice grain-sized dark red nodule on the right side of her face without obvious cause, which gradually increased to the size of a soybean and turned brown. Feeling no subjective symptoms, she did not seek treatment. One week ago, pruritus appeared, so the patient visited our hospital for treatment. She was previously in good health and had no similar family history. The patient had received no prior treatment. Smoking status, family history, and relevant exposures are unknown; Dermoscopy was not performed. The clinical manifestations, pathological features, and immunological test results of the patient are demonstrated in Figures 1–7.

### 2.1 Physical examination

No abnormalities were detected upon systemic examination. Dermatological findings: A soybean-sized brown nodule was visible in the right preauricular area, which had a clear boundary and bulged on the skin surface. The nodule surface was smooth without ulceration, and telangiectasia was seen. The texture was moderate and there was no tenderness.

### 2.2 Laboratory findings

No abnormalities were found upon routine blood and urine tests, coagulation function test and syphilis/HIV screening. Chest X-ray and abdominal B-ultrasound also revealed no abnormalities. Cutaneous histopathology: epidermal processes disappeared, and the dermis was diffusely infiltrated by substantial tumor cells that were in the nested or clumpy form. These polygonal tumor cells were round or oval, which had abundant cytoplasm filled with eosinophilic granules, and were stained pale pink. The cellular nuclei were small, hyperchromatic and centered, with few mitotic figures, and multiple nuclei were observed in some tumor cells. Immunohistochemistry: S-100 (+), CD56 (−), NSE (−), vimentin (+), CD68 (+), Ki-67 (+), cell proportion: 5%, p53 (−), SAM (+−). CK (−), calponin (−), CD163 (−), desmin (−), CD34 (−), CD45 (−), CD30 (−), SMA (1A4) (−), ALK (−), HMB45 (−), melan-A (−).

The mark “(+)” represents a positive immunohistochemistry result. The mark “( + ⁣−) represents a focally/weakly positive immunohistochemistry result. The mark “(−)” represents a negative immunohistochemistry result. The diagnostic significance of these negative immunohistochemical findings lies in their ability to differentiate granular cell tumors from potential mimickers, including gastrointestinal stromal tumors, smooth muscle tumors, anaplastic large cell lymphoma, and melanoma.

### 2.3 Diagnosis

Cutaneous granular cell tumor.

#### 2.3.1 Cutaneous lesion characteristics

A soybean-sized brown nodule was visible in the right preauricular area.

#### 2.3.2 The histopathological features

The dermis was diffusely infiltrated by substantial tumor cells that were in the nested or clumpy form. These polygonal tumor cells were round or oval, which had abundant cytoplasm filled with eosinophilic granules.

#### 2.3.3 The immunohistochemical results

S-100 (+), vimentin (+), CD68 (+), Ki-67 (+), cell proportion: 5%, SAM (+ −). Immunohistochemistry rules out melanoma, tumors of lymphoid origin, smooth muscle tumors, keratoacanthoma, SCC, xanthoma, and dermatofibroma, etc.

### 2.4 Treatment and prognosis

Complete excision of the mass, undertaken under local infiltration anesthesia, was achieved with a circumferential margin of 0. 5 cm. The long-term prognosis remains to be determined through ongoing follow-upNo recurrence was found during the 1-year follow-up, and the follow-up is ongoing.

This case report adheres to the CARE guidelines.

## 3 Discussion

As a rare tumor formerly known as granular cell myoblastoma, the granular cell tumor (GCT) was first reported by Abrikossoff in 1926 (2), which is named for the granular shape of tumor cells. Granular cell tumors most frequently affect women between 40 and 60 years of age and are common on the head and limbs, but reports of their occurrence on facial skin are rare. We report a case of a solitary GCT on the face of a 32-year-old female. The nodule was clinically notable for its potential to be misdiagnosed as an epidermal cyst, adnexal neoplasm, melanoma, dermatofibroma, or basal cell carcinoma. This case underscores the importance of considering GCT in the differential diagnosis of solitary facial nodules. GCTs occurring in the skin are mostly painless skin nodules, and the patients are generally asymptomatic.

Granular cell tumors most frequently occur in the mucosa, breast, and skin, with a relatively high incidence in the digestive tract. The esophagus is the most commonly affected site, while in the oral cavity, the tongue high nce on facial skin are rare. We report a case of a solitary GCT on the face of a 32-year-oldlso be found in the floor of the mouth, upper lip, and lower lip. Most tumors present as solitary, painless, yellow or pink nodules on the anterior tongue (3). Unlike granular cell tumors in other sites, S-100-negative granular cell tumors have been identified in the oral cavity, which are considered to be of non-neural origin (4).

The majority of GCTs are benign, while aggressive malignant GCTs account for only 1%–he majority of GCTs are benign (5), which are characterized by nuclear pleomorphism, tumor cell spindling, vesicular nuclei with large nucleoli, increased nuclear-to-cytoplasmic ratio, necrosis, and increased mitotic rate (>2 mitoses/10HPF) (1). Ki-67, a nuclear antigen specifically expressed in proliferating cells, serves as a proliferation index. Its expression level differs significantly between benign and malignant soft tissue tumors (6). A Ki-67 index of <10% indicates that most tumor cells are quiescent with slow proliferation. Some authors have demonstrated that the upregulation of both p53 and Ki-67 is well correlated with an aggressive clinical course and malignant behavior in granular cell tumors. The origin of GCT remains controversial, with most research results implying its derivation from the neural tissue or Schwann cells (7). In this case, the tumor was positive for S-100, indicating a neural origin. Nevertheless, there are also case reports of non-neuronal GCTs of the skin (8, 9). Compared to the traditional GCTs, the non-neuronal type lacks the expression of S-100 and can show stronger nuclear atypia and mitotic activity.

Diagnosis of GCTs is based primarily on the histopathological examination. Regarding the histopathological features of GCTs, the lesions are located mainly in the dermal, subcutaneous or submucosal tissue, and the tumor cells mostly exhibit banded or nested growth. The tumor cells are large, polygonal and filled with fine eosinophilic granules in the cytoplasm. The cellular nuclei are small and centered, which are either round or oval. Common immunohistochemical markers: S-100 protein (+), NSE (+), CD68 (+), CK (−), SMA (−), GFAP (−), etc.

The skin lesion of the patient in this report was a solitary painless firm nodule on the face. Base on the histopathological findings and immunohistochemical results, she was diagnosed with granular cell tumor. Clinically, this disease needs to be differentiated from keratoacanthoma, nodular squamous cell carcinoma, nodular xanthoma, dermatofibroma, nodular basal cell carcinoma, malignant granular cell tumor, rhabdomyomas and leiomyomas. Currently, GCTs are treated chiefly by surgical resection, and the prognosis is good when no residual tumor cells are left at the surgical margin, although close follow-up is still required.

## Data Availability

The raw data supporting the conclusions of this article will be made available by the authors, without undue reservation.
